# Baum Test as an Adjunctive Monitoring Tool in a Case of Major Depressive Disorder With Prominent Anhedonia

**DOI:** 10.7759/cureus.116805

**Published:** 2026-09-23

**Authors:** Kunio Maekubo, Jumpei Maruta

**Affiliations:** 1 Neuropsychiatry, Osaka Metropolitan University, Osaka, JPN

**Keywords:** anhedonia, baum test, major depression, major depressive disorder, monitoring tool, projective test, tree drawing test

## Abstract

Anhedonia is a core symptom of major depressive disorder associated with poor prognosis and treatment resistance. Assessment often relies on subjective self-reports and clinician-rated scales, and discrepancies between these evaluations can complicate clinical interpretations. The Baum test, a projective tree-drawing test, has recently been examined using quantitative indices such as the number of occupied areas on a page, to allow a more objective assessment. Here, we report the case of a 58-year-old woman with major depressive disorder characterized by prominent anhedonia. Upon admission, evaluations demonstrated a marked discrepancy between the patient’s self-rated depression score, which indicated moderate to severe symptoms, and the clinician-rated scale, which suggested only mild depression. During electroconvulsive therapy, the Baum test was repeatedly administered, and the number of occupied areas appeared to change in parallel with clinical improvement and subsequent symptom fluctuations. These findings suggest that the quantitative indices of the Baum test may reflect changes in motivational or behavioral engagement that are not fully captured by conventional rating scales. These findings serve as a hypothesis-generating observation suggesting that the Baum test may serve as a simple, noninvasive adjunctive tool for longitudinal symptom monitoring, though its interpretation should consider cognitive influences and future studies are needed to establish its diagnostic validity.

## Introduction

Major depressive disorder (MDD) is a prevalent and debilitating psychiatric condition characterized by persistent depressed mood, loss of interest or pleasure, and various cognitive and somatic symptoms [1]. According to the Diagnostic and Statistical Manual of Mental Disorders, Fifth Edition, Text Revision (DSM-5-TR), a diagnosis of MDD requires the presence of at least five out of nine core depressive symptoms during two weeks, representing a change from previous functioning, with at least one symptom being either depressed mood or loss of interest or pleasure [1]. Among these symptoms, anhedonia is a core symptom of MDD associated with impaired pleasure and dysfunctional reward processing [2,3]. Anhedonia has been linked to treatment resistance and a poor prognosis, and requires careful assessment and targeted treatment strategies [4,5]. However, its evaluation primarily depends on subjective self-reports and clinical interviews. Although objective biomarkers such as pupillometry and acoustic analysis, as well as biological signatures of the inflammatory/immuno-metabolic subtype (e.g., elevated C-reactive protein (CRP), altered kynurenine pathway metabolites, and disrupted corticostriatal reward circuitry), have been extensively investigated [6], no widely accepted tests are available for routine clinical practice. When discrepancies arise between subjective self-evaluation and clinician-rated assessments, obtaining an accurate understanding of a patient’s internal state poses a clinical challenge.

To address this challenge and evaluate depressive symptoms and subjective states thoroughly, various clinical scales and psychological tools are utilized. In this case report, we employed a combination of self-report and observer-rated scales as well as a projective measure: the Self-Rating Depression Scale (SDS; Zung, 1965) [7], a 20-item self-report questionnaire developed to quantify depressive severity based on a four-point Likert scale (score range: 20-80, with higher scores indicating greater severity); the Hamilton Depression Rating Scale (HAM-D; Hamilton, 1960) [8], a clinician-administered 17-item scale designed to assess objective symptom severity based on a mix of three-point and five-point rating criteria (scores ≥8 generally indicate depression); and the Baum Test (Tree Drawing Test; Koch, 1952) [9], a projective psychological assessment created by Karl Koch that evaluates non-verbal expression, emotional states, and underlying personality structure through qualitative interpretation of drawn tree characteristics (e.g., trunk structure, roots, and foliage).

The Baum test has been used to screen for depressive states [9-11]. Although traditional interpretation requires specialized training, recent studies have demonstrated that quantitative indices such as tree height, width, and the number of occupied areas on a page allow for an objective evaluation [10-13]. In particular, the number of occupied areas have been useful in detecting depressive states without requiring complex interpretive skills [13]. Among these quantitative indices, the number of occupied areas was selected in this case because it directly reflects spatial engagement and activity level without requiring intricate measurements, making it a pragmatic choice for serial monitoring. Conversely, because the number of occupied areas is also influenced by attention and concentration, previous studies have reported that reduced cognitive function is associated with a decrease in the number of occupied areas and a simplification of tree drawings [14-16]. Given that treatments such as electroconvulsive therapy (ECT) can be accompanied by transient cognitive impairment [17], the potential influence of cognitive function warrants consideration when interpreting changes in this index over the clinical course.

Here, we report the case of a patient with MDD with prominent anhedonia, in whom a marked discrepancy was observed between subjective and clinician-rated symptom scales. The Baum test was administered repeatedly during treatment and changes in the number of occupied areas were examined in relation to the clinical course.

## Case presentation

The patient was a 58-year-old married woman and a university graduate. Her psychiatric family history was unremarkable, though her father had a history of pancreatic cancer and her mother had breast cancer. She had no past physical illnesses, psychiatric hospitalizations, or substance abuse. Her developmental and social history was unremarkable, with a history of good interpersonal relationships and no interpersonal conflicts from childhood. Her premorbid personality was described as conscientious and meticulous, and her activities of daily living were fully independent. After graduating from university, she worked in an office until marrying at age 24, at which point she became a homemaker. She currently resides with her husband, has two independent daughters, and maintains good relationships with her family. At the age of 27 years, she visited a hospital due to depressed mood and anxiety and was diagnosed with depression and anxiety neurosis. Her symptoms remitted within approximately six months, and she discontinued follow-up without recurrence. Because detailed medical records from the first hospital were unavailable, the specific medication names, dosages, duration of pharmacotherapy, and whether medications were continued until the discontinuation of follow-up remain unknown.

Three years before the current admission, she developed depressive symptoms, including anxiety about COVID-19, fatigue, insomnia, and loss of appetite, and visited a psychiatric clinic. She was diagnosed with depression. The specific diagnostic criteria and psychometric testing used at the clinic were not documented in the available records. Combination therapy with escitalopram, duloxetine, and sulpiride was initiated. Although the exact daily dosages for these initial medications remain unknown due to a lack of precise records, she achieved remission seven months later. Antidepressants were tapered and discontinued over three months at the patient’s request. Five months following the discontinuation of medication, her symptoms recurred without an obvious trigger. Escitalopram and duloxetine were restarted; however, due to an inadequate response, duloxetine was discontinued and augmentation therapy with aripiprazole was initiated. After titration of aripiprazole up to 1.5 mg/day, her symptoms remitted within six months. While the exact dosages for escitalopram and duloxetine during this second trial remain unknown, the pharmacotherapy was maintained. However, three months later, she again developed depression symptoms and loss of interest. Despite treatmet with two antidepressants, combination therapy, and augmentation strategies, she continued to report, “I cannot enjoy anything,” and her anhedonia persisted. Specifically, combination therapy with escitalopram (20 mg/day) and duloxetine (40 mg/day) was administered, but yielded insufficient response. Escitalopram was then switched to paroxetine (40 mg/day). Each antidepressant trial was maintained for at least four weeks. An attempt to titrate duloxetine to 60 mg/day induced tachycardia, requiring a dose reduction back to 40 mg/day. Regarding augmentation strategies, aripiprazole was increased up to 3 mg/day without adequate response, and subsequently switched to brexpiprazole (0.5 mg/day), which also failed to improve her clinical state. These augmentation strategies were evaluated for at least four weeks at maximum tolerated doses. She was admitted to the psychiatric department of our hospital after 11 months of unremitting symptoms for electroconvulsive therapy (ECT). The patient’s pre-admission depressive episodes and relapse course are summarized in Table 1.

**Table 1:** Pre-admission depressive episodes and relapse course ECT: electroconvulsive therapy.

Episode	Age / timing at onset	Duration	Triggers / main symptoms	Treatment and course
First episode	Age 27	Approx. 6 months	Depressed mood, anxiety	Treated at a hospital; remitted in approximately 6 months (medication details unavailable).
Second episode	3 years before admission	7 months	COVID-19-related anxiety, fatigue, insomnia, loss of appetite	Pharmacotherapy at a psychiatric clinic (escitalopram, duloxetine, and sulpiride; exact doses unknown); remitted in 7 months, followed by a 3-month taper and discontinuation.
Third episode	Approx. 2 years before admission	6 months	Relapse without an obvious trigger	Escitalopram and duloxetine restarted, then aripiprazole augmentation (up to 1.5 mg/day); remitted within 6 months with continued pharmacotherapy.
Fourth episode (index)	Approx. 1 year before admission	11 months (refractory)	Prominent anhedonia (“I cannot enjoy anything”)	Multiple antidepressant trials and augmentation (including aripiprazole and brexpiprazole) without adequate response; admitted for ECT.

Before admission, the patient reported that television viewing, which had previously been part of her daily routine and a source of relaxation, had gradually lost appeal. Although she continued to habitually sit in front of the television, she stated that she “felt nothing” while watching programs and was unable to follow storylines with interest. She denied experiencing intense sadness during these moments but described a pervasive absence of enjoyment. Family members observed that she no longer commented on programs or expressed curiosity about upcoming episodes.

On admission, a comprehensive mental status examination was conducted. The patient was alert and oriented. Her speech was fluent but soft, with a reduced tone. Her affect was restricted and blunted, corresponding to a severely depressed mood. No formal thought disorder, delusions, or hallucinations were observed. Psychomotor retardation was mild, but she exhibited a prominent loss of interest and motivation. At the time of admission, her prescribed regimen consisted of paroxetine (40 mg/day), duloxetine (40 mg/day), and ethyl loflazepate (0.5 mg/day). Paroxetine and duloxetine were maintained throughout hospitalization and administered concurrently with ECT, whereas ethyl loflazepate was discontinued on hospital day eight in preparation for ECT. No formal psychotherapy was provided during the hospital stay.

Her Self-rating Depression Scale (SDS) [7] score was 60, indicating moderate to severe depression, whereas the Hamilton Depression Rating Scale (HAM-D) [8] score was 11, indicating mild depression and demonstrating a discrepancy between the subjective and clinician-rated assessments. The initial Baum test was administered on admission. As part of the standardized administration, the patient was provided with a blank sheet of A4 paper, a pencil, and an eraser, and was instructed simply to “draw a tree.” The number of occupied areas was quantified using a transparent A4-sized grid overlay divided into 20 rows and 14 columns (totaling 280 grid cells), which was placed directly over the drawn tree, and the number of grid cells containing any part of the tree drawing was counted [13] by a rater blinded to the patient’s concurrent SDS and HAM-D scores. To ensure measurement reliability, all drawings across all timepoints were assessed by this same rater, and repeated counts were performed on two separate occasions to evaluate intra-rater reliability, which confirmed high consistency across measurements.

The Baum test administered on admission revealed that the number of occupied areas (occupied squares) was 62. On day seven of hospital admission, her Mini-Mental State Examination (MMSE) [18] score, assessed using the Japanese version (MMSE-J) [19], was 28, indicating normal cognitive function. The administration of the SDS, HAM-D, MMSE-J, and Baum test was performed as part of routine clinical care for evaluating and monitoring the patient's depressive symptoms during hospitalization. The grid-based quantification of occupied areas, together with the blinded and repeated scoring described above, was performed retrospectively after discharge for the purposes of preparing this case report, and did not influence real-time clinical management.

Physical and neurological examinations, including vital signs, revealed no significant abnormal findings upon admission. The laboratory investigations at admission are summarized in Table 2.

**Table 2:** Laboratory investigations at admission

Laboratory parameter	Result	Reference range	Unit
White blood cells (WBC)	6,300	3,300–8,600	/µL
Red blood cells (RBC)	4.24	3.86–4.92	×10⁶/µL
Hemoglobin (Hb)	13.9	11.6–14.8	g/dL
Platelets (PLT)	33.6	15.8–34.8	×10⁴/µL
C-reactive protein (CRP)	0.01	0.00–0.14	mg/dL
Total bilirubin (T-Bil)	0.78	0.40–1.50	mg/dL
Albumin (Alb)	4.5	4.1–5.1	g/dL
Aspartate aminotransferase (AST)	32	13–30	U/L
Alanine aminotransferase (ALT)	53	7–23	U/L
Urea nitrogen (UN)	13	8–20	mg/dL
Creatinine (Cr)	0.63	0.46–0.79	mg/dL
Sodium (Na)	140	138–145	mEq/L
Potassium (K)	3.7	3.6–4.8	mEq/L
Blood sugar (BS)	107	73–109	mg/dL
Free triiodothyronine (FT3)	3.62	2.30–4.00	pg/mL
Free thyroxine (FT4)	1.35	0.90–1.70	ng/dL
Thyroid-stimulating hormone (TSH)	4.23	0.50–5.00	µIU/mL

Most values were within institutional reference ranges, with mild elevations of aspartate aminotransferase (AST) and alanine aminotransferase (ALT) that were not considered suggestive of an organic cause of the psychiatric presentation. Imaging studies and electroencephalography after admission similarly revealed no abnormalities that would suggest an organic etiology. Prior to initiating ECT, a clinical and pre-anesthetic safety assessment was performed, and no findings contraindicating general anesthesia or ECT were identified. Standard pre-ECT preparation and safety procedures were implemented. ECT was administered twice weekly for a total of six sessions on hospital days 16, 20, 23, 27, 30, and 34. As noted, paroxetine (40 mg/day) and duloxetine (40 mg/day) were continued alongside ECT.

On day 15, immediately before ECT initiation, the Baum test revealed 55 occupied areas. On day 21, after the second session (administered on day 20), the SDS and HAM-D scores decreased to 50 and 5, respectively, and the number of occupied areas increased to 105. Depression, guilt, anxiety, sadness, and fatigue were almost completely resolved. On day 28, after the fourth session (administered on day 27), her activity level (including walking around the ward) increased, and her loss of interest improved. The SDS score decreased to 33, the HAM-D score to 0, and the number of occupied areas increased to 100. The fifth and sixth ECT sessions were completed on days 30 and 34, respectively.

However, on day 35 (the day after the sixth ECT session), shortly before discharge, the patient reported anxiety about returning home and her symptoms fluctuated. The SDS score increased to 50, the HAM-D score remained 0, and the number of occupied areas decreased markedly to 30. The MMSE score was 25. Her complaints of anxiety regarding returning home subsided the following day, and she was discharged on schedule on day 37 without any changes to her medication regimen (paroxetine 40 mg/day and duloxetine 40 mg/day). Following discharge, the patient was referred back to another clinic for ongoing outpatient care. No post-discharge follow-up was conducted at our hospital; therefore, subsequent symptoms, quality of life, psychological assessments (including repeat Baum testing), and pharmacological adjustments remain unknown.

Written informed consent was obtained from the patient for publication of this case report and any accompanying images.

All laboratory values were within normal limits except for mild elevations in AST and ALT. Imaging studies and electroencephalography likewise showed no abnormalities suggestive of an organic etiology. Baum test drawings obtained at five representative timepoints during hospitalization are shown in Figure 1.

**Figure 1:**
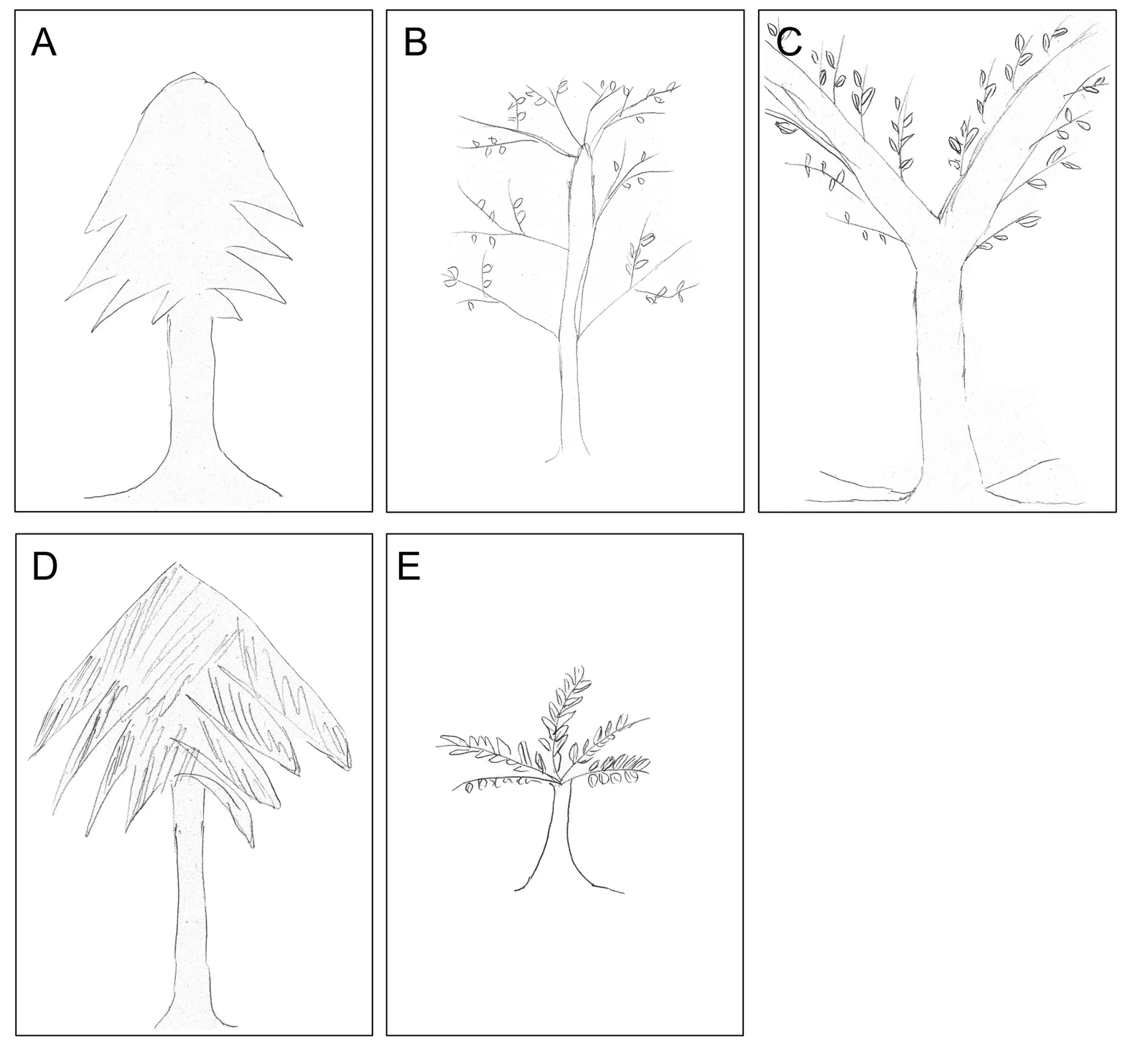
Sequential changes in the Baum test during hospitalization (A) Day 1 (at admission); (B) Day 15 (before initiation of electroconvulsive therapy or ECT); (C) Day 21 (after the second ECT session); (D) Day 28 (after the fourth ECT session); (E) Day 35 (pre-discharge).

The overall clinical course, including changes in SDS, HAM-D, the number of occupied areas, and MMSE, is summarized in Figure 2 and Table 3.

**Figure 2:**
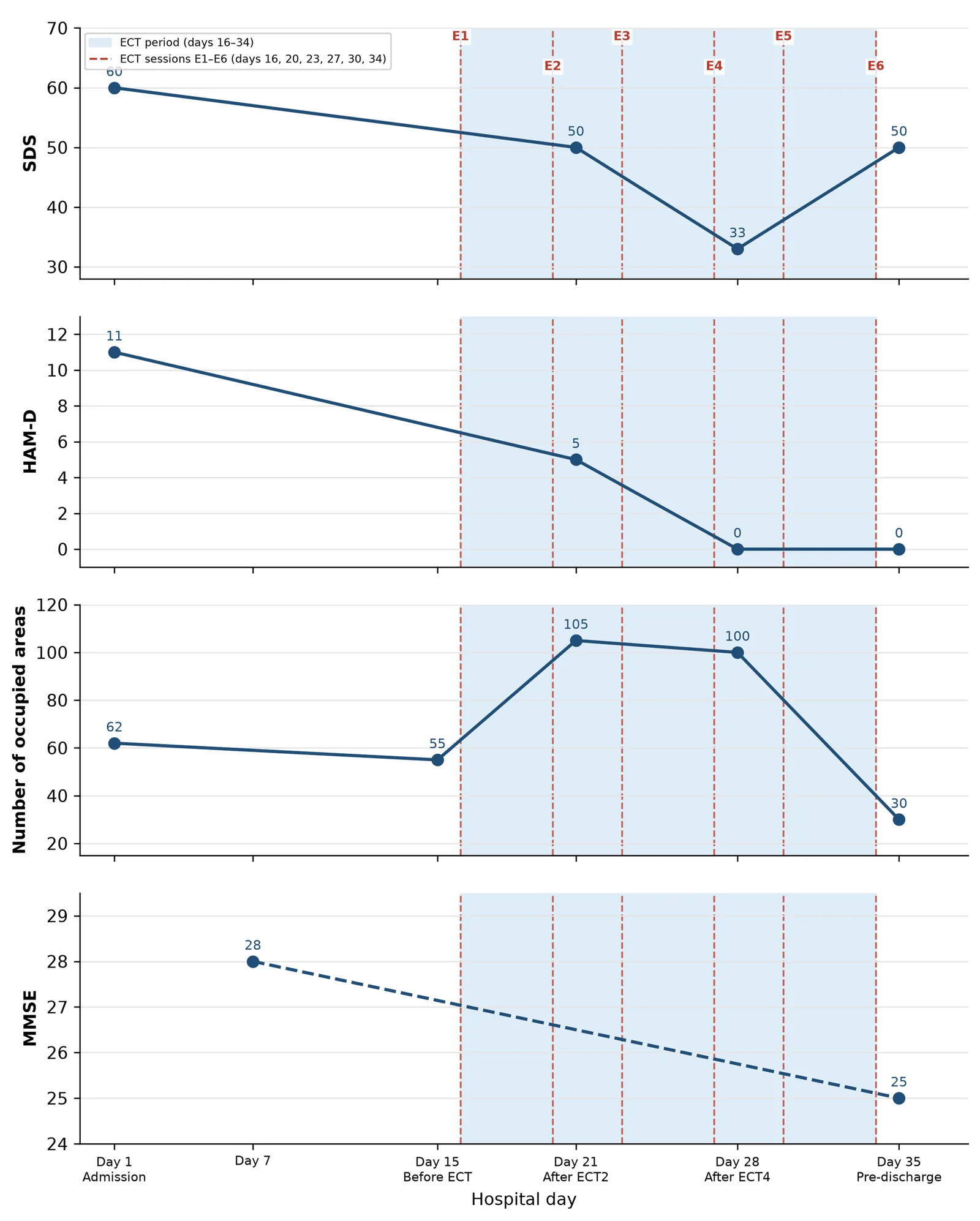
Clinical course and changes in psychological and cognitive assessment scores during hospitalization Vertical panels share a common hospital-day timeline. The shaded band indicates the electroconvulsive therapy or ECT period (days 16–34). Red vertical dashed lines with labels E1–E6 mark each ECT session (days 16, 20, 23, 27, 30, and 34). (a) Self-rating Depression Scale (SDS) scores; (b) Hamilton Depression Rating Scale (HAM-D) scores; (c) Number of occupied areas in the Baum test; (d) Mini-Mental State Examination (MMSE) scores, measured on day 7 and day 35.

**Table 3:** Serial assessment scores during hospitalization SDS: Self-rating Depression Scale; HAM-D: Hamilton Depression Rating Scale; MMSE: Mini-Mental State Examination; ECT: electroconvulsive therapy. Em dashes (—) indicate that the measure was not administered at that timepoint.

Hospital day	Clinical context	SDS	HAM-D	Occupied areas	MMSE
Day 1	Admission	60	11	62	—
Day 7	Cognitive assessment	—	—	—	28
Day 15	Before initiation of ECT	—	—	55	—
Day 16	ECT session 1	—	—	—	—
Day 20	ECT session 2	—	—	—	—
Day 21	Assessment after the second ECT session	50	5	105	—
Day 23	ECT session 3	—	—	—	—
Day 27	ECT session 4	—	—	—	—
Day 28	Assessment after the fourth ECT session	33	0	100	—
Day 30	ECT session 5	—	—	—	—
Day 34	ECT session 6	—	—	—	—
Day 35	Pre-discharge assessment	50	0	30	25

## Discussion

A marked discrepancy between subjective and clinician-rated assessments existed on admission, demonstrated by the patient’s SDS score indicating moderate-to-severe symptoms and the clinician-rated HAM-D score suggesting mild depression. In cases in which anhedonia predominates, externally observable depressive signs such as psychomotor retardation, agitation, or pessimistic rumination may be less conspicuous [15]. Consequently, discrepancies can arise between subjective reports of patient suffering and clinician-rated scales that rely heavily on observable features. In some clinical contexts, such discrepancies may lead clinicians to unintentionally question whether symptoms are overestimated. However, conventional rating scales may insufficiently capture the experiential dimensions of diminished pleasure and motivation [20].

Projective drawing methods are non-verbal psychological assessment tools in which individuals project their internal emotional states, unconscious conflicts, and psychological dynamics onto visual representations, such as drawing trees or figures [9]. Because these projective drawings, including the Baum test, elicit spontaneous responses without fixed, self-descriptive options, quantitative indices such as the number of occupied areas may be less susceptible to intentional symptom-report bias than self-report scales [9]. Regarding the choice of quantitative indices, the number of occupied areas was adopted rather than linear single parameters such as tree height or width. Previous studies applying the grid-based area counting method have noted that this approach provides clear operational definitions, minimizing subjective interpretation and inter-rater variability [13].

By directly reflecting the patient’s overall spatial engagement and activity level without requiring intricate calculations, this practical quantitative metric serves as a feasible tool for continuous serial monitoring. In the present case, although it is difficult to conclude that the observed discrepancy was specific to anhedonia, the parallel change in the number of occupied areas with clinical improvement suggests that this quantitative index may have reflected aspects of the patient’s internal motivational or behavioral engagement that were not fully detected by clinician-rated scales. Because no established normative cutoff exists for the number of occupied areas, this index was interpreted as a relative, within-subject measure of change rather than as an absolute severity marker. Compared to previous studies using the Baum test for depression screening, where reduced occupied areas or tree size served as cross-sectional screening markers [10,11,13], our findings extend its exploratory clinical utility to longitudinal monitoring. The marked reduction in the number of occupied areas on day 35, despite a HAM-D score of 0, further illustrated the potential dissociation between the clinician-rated severity and non-verbal quantitative indices.

The influence of cognitive function should also be considered when interpreting these quantitative indices. Previous studies have consistently reported that reduced cognitive function is associated with a decrease in the number of occupied areas and a simplification of tree drawings, and the Baum test has accordingly been investigated as a screening tool for Alzheimer’s disease and mild cognitive impairment [14-16]. Given this established direction of association, a decline in cognitive function would be expected to decrease, rather than increase, the number of occupied areas. The marked increase observed after the second ECT session (55 to 105) is therefore unlikely to be explained by cognitive decline and may reflect changes in psychological state or motivational engagement. In contrast, the subsequent marked decrease on day 35 (100 to 30) - assessed the day after the sixth ECT session (day 34) and shortly before anticipated discharge - is directionally consistent with a possible contribution of transient cognitive impairment following ECT [17]. However, because the HAM-D score remained 0 and the MMSE score showed only a slight, clinically insignificant decrease (28 to 25) from baseline, this slight decline in MMSE may reflect mild transient post-ECT cognitive fluctuations or, alternatively, heightened internal distress and anxiety related to the upcoming discharge.

Thus, while a transient post-ECT effect on drawing performance cannot be completely ruled out, the pre-discharge drop in occupied areas may have been influenced by psychological distress related to anticipated discharge rather than substantial cognitive impairment. Taken together, these observations suggest that cognitive fluctuation alone is unlikely to fully account for the changes in the number of occupied areas observed in this case. Nevertheless, because the MMSE was assessed only on day seven and day 35, cognitive function during the intervening period was not directly measured, and this interpretation remains inferential.

A major strength of this report lies in the continuous longitudinal monitoring of quantitative drawing indices during ECT, which provided objective non-verbal data during a period of marked subjective-objective symptom discrepancy. In clinical settings where verbal self-report is limited or discrepant from clinician ratings, such adjunctive quantitative indices may offer complementary information; however, inconsistent predictor selection across studies and the lack of standardized cutoffs currently constrain broad clinical implementation.

This report has several limitations. First, as a single case report, the generalizability of these findings is limited, and the relationship between the number of occupied areas and anhedonia warrants confirmation in larger samples. Second, because the Baum test was administered repeatedly within a short period, a practice effect independent of clinical state cannot be entirely excluded. Third, the number of occupied areas was assessed by a single rater, and inter-rater reliability was not evaluated. Fourth, validated anhedonia-specific rating scales (e.g., the Snaith-Hamilton Pleasure Scale or the Dimensional Anhedonia Rating Scale) were not administered in this case [20], limiting direct conclusions regarding the relationship between Baum test changes and specific hedonic mechanisms. Future research should explore these quantitative indices in larger prospective cohorts, ideally with standardized administration procedures and validated anhedonia measures, to clarify their clinical utility.

## Conclusions

In patients with MDD with prominent anhedonia, discrepancies between subjective and clinician-rated assessments may complicate the evaluation of treatment response. In this patient’s case, the number of occupied areas observed in the Baum test appeared to change in parallel with clinical improvement and subsequent symptom fluctuations, suggesting that the test may serve as a simple and non-invasive adjunctive tool for monitoring motivational or behavioral engagement related to depressive symptoms. Combining quantitative drawing indices with conventional clinical scales may potentially contribute to more comprehensive longitudinal monitoring of psychiatric symptoms. However, the interpretation of Baum test results must include the potential influence of cognitive function, particularly in patients undergoing treatments such as ECT. Prospective studies with larger samples and validated anhedonia measures are required before clinical implementation can be broadly recommended.
